# Efficacy of Probiotic Supplements on Brain-Derived Neurotrophic Factor, Inflammatory Biomarkers, Oxidative Stress and Cognitive Function in Patients with Alzheimer’s Dementia: A 12-Week Randomized, Double-Blind Active-Controlled Study

**DOI:** 10.3390/nu16010016

**Published:** 2023-12-20

**Authors:** Yu-Chieh Hsu, Yen-Yu Huang, Shin-Yu Tsai, Yi-Wei Kuo, Jia-Hung Lin, Hsieh-Hsun Ho, Jui-Fen Chen, Ko-Chiang Hsia, Yu Sun

**Affiliations:** 1 Department of Research and Design, Glac Biotech Co., Ltd., Tainan 744, Taiwan; yuchieh.hsu@glac.com.tw (Y.-C.H.); shin-yu.tsai@glac.com.tw (S.-Y.T.); vic.kuo@glac.com.tw (Y.-W.K.); jiahung.lin@glac.com.tw (J.-H.L.); sam.ho@glac.com.tw (H.-H.H.); juifen.chen@glac.com.tw (J.-F.C.); shawn.hsia@glac.com.tw (K.-C.H.); 2 Department of Neurology, En Chu Kong Hospital, New Taipei City 237, Taiwan; 3 Department of Neurology, National Taiwan University Hospital, College of Medicine, National Taiwan University, Taipei 100, Taiwan

**Keywords:** Alzheimer’s disease, probiotics, brain-derived neurotrophic factor (BDNF), cognitive function, oxidative stress, anti-inflammation, clinical trial

## Abstract

The role of neurotrophic factors, oxidative stress, and inflammation in the pathogenesis of Alzheimer’s disease (AD) has been explored. Animal studies have reported the positive effects of probiotics on these factors. Some clinical studies also support the potential role of probiotics in improving cognitive function via the gut–brain axis in older adults. However, clinical experimental studies evaluating the efficacy of probiotics targeting the neurotrophic factors and inflammatory biomarkers, particularly among AD patients, remain very limited. In this randomized, double-blinded, active-controlled trial, we used multi-strain probiotic supplements, including *Bifidobacterium longum* subsp. *infantis* BLI-02, *B. breve* Bv-889, *B. animalis* subsp. *lactis* CP-9, *B. bifidum* VDD088, and *Lactobacillus plantarum* PL-02 as the intervention. Participants were divided into an active control group (received probiotic supplements containing 5 × 10^7^ colony-forming units per day, CFU/day) and a treatment group (1 × 10^10^ CFU/day). Student’s *t* test was applied as the main method of statistical analysis. After 12 weeks of intervention, the treatment group demonstrated a 36% increase in serum brain-derived neurotrophic factor (BDNF) (* *p* = 0.005), a reduction in IL-1β (* *p* = 0.041), and an increase in antioxidant superoxide dismutase (SOD) (* *p* = 0.012). No significant change was found in the active control group. A trend toward less cognitive deterioration was observed, but not statistically significant. In conclusion, this study presents evidence supporting the benefits of multi-strain probiotics in enhancing BDNF, ameliorating inflammation and oxidative stress in AD patients.

## 1. Introduction

Population aging is a global concern. According to estimations by the United Nations, the global population aged 65 or older will double from 700 million in 2019 to 1.5 billion by 2050. Aging can affect the nervous system, and may lead to cognitive impairments, potentially resulting in dementia. Alzheimer’s disease (AD) is the most common form of dementia and may contribute to 60–70% of cases [1]. AD is an irreversible form of neurodegenerative disorder that leads to cognitive impairment, memory loss, and disruptions in brain function, ultimately affecting daily activities [2]. It is characterized by the deposition of amyloid plaques, the abnormal phosphorylation of tubulin-associated unit (TAU) proteins in the brain, and the degeneration of neurons [3]. With AD being the fifth leading cause of death among individuals aged 65 and above, the economic burden of treating and caring for AD patients is projected to be substantial. By 2022, the projected cost of treating and caring for AD and other dementias in the United States is estimated to reach USD 339.5 billion [4]. Medications commonly used in the medical field for AD include acetylcholinesterase inhibitors (donepezil, rivastigmine, galantamine, and tacrine) [5] and the *N*-methyl-d-aspartate receptor antagonist memantine (used alone or in combination with donepezil) [6], which help to regulate glutamate activity in the brain. While these medications can provide temporary improvements in memory, they do not alter the progression of AD [7].

In recent years, numerous studies have highlighted the relationship between the human gut microbiota and neurodegenerative diseases [8,9,10]. This intricate relationship between the gut microbiome and the central nervous system is commonly referred to as the gut–brain axis. Gut dysbiosis, an imbalance in the gut microbial community, and the disruption of the microbiota–gut–brain axis are believed to contribute to the development and progression of AD [11]. Probiotics, which are beneficial strains of bacteria consumed in sufficient quantities, have gained attention for their potential to promote a healthy gut microbiota [12]. Their effects extend beyond digestive health, as study indicates that probiotics can restore the homeostasis of intestinal microbiota and may have the potential to delay the progression of AD, especially in inflammation and oxidative stress, thereby improving cognitive decline [13]. Brain-derived neurotrophic factor (BDNF), a neurotrophic protein, plays a crucial role in the functioning of degenerating neurons in AD. AD patients exhibit significantly lower serum BDNF levels compared to healthy individuals [14]. Research suggests that a combination of *Lactobacillus* and *Bifidobacterium* can effectively increase BDNF levels in individuals with neurological disorders [15]. A double-blinded clinical trial demonstrated that a probiotic blend containing *Lactobacillus* and *Bifidobacterium* strains significantly improved the cognitive scores of AD patients compared to a placebo group [16].

In addition to the effects on BDNF, probiotics may also have other beneficial effects regarding the inflammation and oxidative stress which contribute to brain degeneration. Our previous study has shown that multi-strains of *Bifidobacterium* and *Lactobacillus* could actively modulate the gut microbiota and increase the levels of short-chain fatty acids (SCFAs) in the serum of middle-aged mice, leading to improved antioxidant activity. Furthermore, the findings confirmed the synergistic effects of live probiotic strains and their metabolites in enhancing antioxidant activity in the brain, liver, heart, and kidney of middle-aged mice [17].

Most of the previous randomized controlled trials using probiotics as intervention were performed either among community-dwelling older adults, or subjects with mild cognitive impairment and dementia without specifically focusing on people clinically diagnosed with AD [18,19,20,21,22,23]. Dementia can be caused by various etiologies, such as brain injury, stroke, drug, or infection. Pathogenesis for those brain insults is quite different from AD, leading to the difficulty to explain the mechanism that underlies the efficacy of probiotic supplements for all types of dementia. In addition, Studies which explore the evidence of the efficacy of probiotics on BDNF, oxidative stress and antioxidant biomarkers using experimental study designs among AD patients are very limited [24,25].

Based on the aforementioned evidence and our previous study, we chose the multi-strain probiotics *B. longum* subsp. *infantis* BLI-02, *B. breve* Bv-889, *B. animalis* subsp. *lactis* CP-9, *B. bifidum* VDD088, and *L. plantarum* PL-02 as part of our study intervention formula, which may have the ability to delay aging and reduce oxidative stress in human beings as that shown in animal studies [17]. The objective of this study was to evaluate the efficacy of these probiotics on BDNF level, biomarkers of oxidative stress and inflammation, and cognitive function in clinically diagnosed AD patients. Additionally, we analyzed alterations in the gut microbiota before and after a 12-week intervention period.

## 2. Materials and Methods

This study, conducted from 2020 to 2022, was a double-blinded, randomized, active-controlled trial evaluating the effects of multi-strain probiotic intervention in patients with AD. The clinical trial received approval from the En Chu Kong hospital’s institutional review board (ECKIRB1080701) and was registered on the US clinical trials website under NCT05145881. All patients or their family members provided signed informed consent before participating.

### 2.1. Participants

To be eligible for inclusion, participants were required to be aged between 50 and 90 years, and to meet the clinical criteria based on any of the following: the Diagnostic and Statistical Manual of Mental Disorders, Fifth Edition criteria (DSM-V) [26]; the National Institute of Neurological and Communicative Disorders and Stroke and the Alzheimer’s Disease and Related Disorders Association (NINCDS-ADRDA Alzheimer’s Criteria) [27]; the National Institute on Aging and Alzheimer’s Association criteria (NI-AAA) (2011) [28]. Patients with mild to moderate dementia with their mental tests, including Taiwanese Mini-Mental State Examination (MMSE) scores ranging from 10 to 25 and Clinical Dementia Rating (CDR) scores of 0.5, 1, or 2, were enrolled. Several criteria were applied to exclude the causes of dementia other than Alzheimer’s disease. Patients with vitamin B12 deficiency, thyroid function abnormalities, positive serum syphilis test, severe metabolic, liver, or kidney dysfunction, as assessed via blood test, were excluded. Additionally, individuals with other potential causes of dementia, such as a history of severe brain trauma, brain tumors, epilepsy, or central nervous system infection, were not included. Patients who had taken immunosuppressive drugs, steroids, antibiotics, or received chemotherapy within the past two weeks were also excluded. Moreover, individuals with psychiatric disorders or severe depression, individuals diagnosed with parkinsonism, alcohol addiction, substance abuse, or those who had consumed probiotic products in the past one month were not included. All participants underwent computed tomography scans or magnetic resonance imaging of the brain to exclude other causes of dementia such as vascular dementia, hydrocephalus, or other brain insults.

### 2.2. Probiotic Strains

All probiotic strains utilized in this study were sourced from Glac Biotech Co., Ltd. (Tainan, Taiwan). The probiotic strains, including *B. longum* subsp. *infantis* BLI-02, *B. breve* Bv-889, and *B. animalis* subsp. *lactis* CP-9, were isolated from breast milk. *B. bifidum* VDD088 was isolated from the healthy infant intestines. Additionally, *L. plantarum* PL-02 was isolated from the fecal sample of Wei-Ling Chen, the renowned gold medalist in the women’s weightlifting at 2008 Olympics.

### 2.3. Intervention and Measurements

All participants were randomly assigned to either the active control group or the treatment group. Each group enrolled 20 participants who were instructed to store probiotic capsules under refrigeration conditions and to take one capsule daily for a period of 12 weeks. To ensure compliance with the treatment, adherence assessments were conducted twice during the study duration. The probiotic capsules of the treatment group contained 1 × 10^10^ CFU/capsule, while the probiotic capsules of active control group contained 5 × 10^7^ CFU/capsule. Each capsule comprised a combination of five probiotic strains, namely BLI-02, Bv-889, CP-9, VDD088, and PL-02, mixed in equal proportions (1:1:1:1:1). During the baseline assessment, participants underwent a comprehensive evaluation that encompassed demographic information, anthropometric data, clinical questionnaires, blood assessments, and stool sampling. After the 12-week intervention period, participants underwent the same set of tests as before.

In the study, the measures included changes in BDNF, and various blood assessments, including cytokines, cortisol, antioxidant enzymes, and peroxides. Additionally, the study involved the use of questionnaires, including MMSE, Alzheimer’s Disease Assessment Scale-Cognitive Subscale (ADAS-Cog), CDR, and Activities of Daily Living Scale (ADL) as part of the cognitive assessment. Furthermore, next-generation sequencing (NGS) was utilized to analyze changes in the composition of gut microbiota.

To ensure unbiased evaluation, the assessment results, as well as any adverse events, were evaluated by an independent physician who was blinded to the patients’ assigned study group.

#### 2.3.1. Assessment of Biochemical Parameters

Blood samples were collected from AD patients and were used to analyze BDNF, cytokine levels (IL-1β and IL-10), cortisol, and oxidative activities (superoxide dismutase (SOD), malondialdehyde (MDA), and protein carbonylation content (PCC)), before and after a 12-week probiotic intervention. The quantification of serum BDNF, IL-1β, and IL-10 was accomplished using enzyme-linked immunosorbent assay (ELISA) kits (BioLegend, Inc., San Diego, CA, USA). Serum cortisol levels were determined using ROCHE Cobas E601/Beckman DxC 800 (Beckman Coulter Inc., Brea, CA, USA). Furthermore, serum levels of SOD, MDA, and PCC were measured using assay kits, following the protocol outlined by Cayman Chemical (Ann Arbor, MI, USA). Subsequently, the samples were measured using an ELISA reader (CLARIOstar^®^ Plus, BMG Labtech, Ortenberg, Germany).

#### 2.3.2. Assessment for Cognitive and Daily Living Function

The MMSE, evaluating orientation to time, orientation to place, registration, attention and calculation, recall, language, repetition, and comprehension of complex instructions, provides a total score ranging from 0 to 30, with a higher score indicating better cognitive function [29]. The CDR is determined through interviews with the family members or main caregivers of participants. The CDR contains six domains for evaluating memory, orientation, judgment and problem-solving, community affairs, home and hobbies, and personal care. The CDR score ranges from 0 (no dementia) to 3 (severe dementia) [30]. The ADAS-Cog provides a comprehensive assessment covering several domains, including word recall, naming objects and fingers, following commands, constructional praxis, ideational praxis, orientation, word recognition task, remembering test directions, spoken language, comprehension, and word-finding. ADAS-Cog yields a total score ranging from 0 to 70, with a higher score indicating worse cognitive function [31]. The ADL test assesses essential tasks necessary for daily functioning with 23 items comprising 6 basic activities of daily living items and 17 instrumental activities of daily living items. The scale yields a total score ranging from 0 to 78, with lower scores indicating increased dependency and challenges in daily living [32]. These assessments were performed by a certificated clinical psychologist before and after the intervention.

#### 2.3.3. Fecal DNA Extraction and NGS Analysis

Fecal samples were collected from participants using the fecal collection tube at week 0 and week 12, and immediately stored at −80 °C. Bacterial DNA was extracted from the fecal samples, which were warmed up to room temperature, using the QIAamp Fast DNA Stool Mini Kit (Qiagen, Hilden, Germany) with certain modifications to the standard protocol. The fecal sample underwent centrifugation at 13,200 rpm for 10 min to eliminate the storage buffer, followed by lysis using Inhibit EX buffer. After homogenization, proteinase K and ethanol were added to the sample to obtain the processed supernatant. Subsequently, the supernatant was subjected to washing with a QIAamp spin column and elution with elution buffer. The concentration of extracted DNA was assessed using NanoDrop 2000 (Thermo Fisher Scientific Inc., Waltham, MA, USA), and a 10× dilution was performed with elution buffer.

The gut microbiome library was generated using the standard V3–V4 region of the 16S rRNA gene. Polymerase chain reaction (PCR) amplification was performed with KAPA HiFi HotStart ReadyMix (Roche, Basel, Switzerland), and the resulting PCR products were purified using AMPure XP magnetic beads (Beckman Coulter Inc., Brea, CA, USA). The quality and quantity of the amplified PCR products were assessed using a Fragment Analyzer (Advanced Analytical, Orangeburg, NY, USA) and a Qubit 3.0 Fluorometer (Thermo Fisher Scientific Inc., Waltham, MA, USA), respectively. Subsequently, the constructed library was sequenced on a MiSeq platform (Illumina, San Diego, CA, USA) with paired-end reads (2 × 301 nt). Each sample was sequenced to generate at least 100,000 reads.

Following data acquisition, the raw paired-end reads were subjected to trimming in accordance with the GreenGene database Version 13.8. Subsequently, the reads that successfully met the quality filters were allocated to operational taxonomic units (OTUs) based on a 97% similarity threshold. Sequences that appeared as singletons or failed to map to the database were removed from the dataset. Taxonomic classification was then performed following the annotation of OTUs. Alpha diversity, beta diversity, and top 7 phyla were employed. Alpha diversity (Chao1) analysis assessed the species complexity within each sample, while beta diversity (Bray–Curtis) analysis evaluated the distinctions among microbial communities.

### 2.4. Statistical Analysis

The gender distribution, as indicated by the numbers, was analyzed using Pearson’s chi-squared test. Continuous variables were presented as mean ± standard deviation or odds ratio (OR) with a 95% confidence interval and were assessed using Student’s *t*-test with IBM SPSS Statistics Version 22.0 (IBM Co., Armonk, NY, USA). Statistical significance was defined as a *p*-value less than 0.05.

## 3. Results

### 3.1. Participant Recruitment

**Figure 1:**
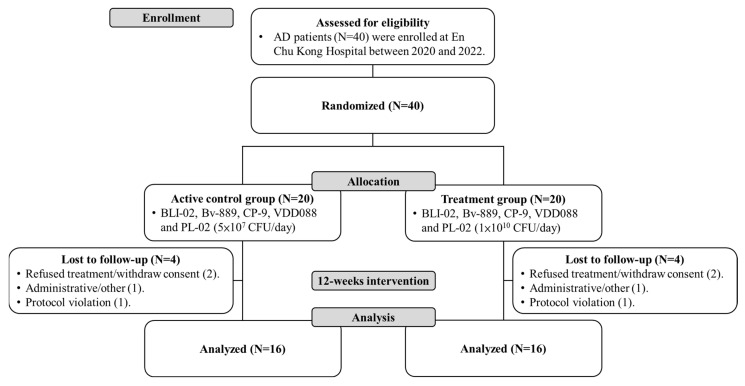
Clinical flowchart of AD patient enrollment.

A total of 40 participants with AD who underwent screening were enrolled in this study at En Chu Kong hospital. All the participants have concomitant use of medications, including acetylcholinesterase inhibitors (donepezil or rivastigmine) and/or the *N*-methyl-d-aspartate receptor antagonist memantine as their standard treatment for AD. They were all randomly assigned to either the active control group (N = 20) or the treatment group (N = 20) during the allocation stage. In each group, four participants who were lost to follow-up because of refused treatment/withdraw consent (two patients), administrative/other (one patient), and protocol violation (one patient), resulting in each group comprising sixteen participants who successfully completed the trial and had complete data for analysis (Figure 1). The average age for the active control group and the treatment group was 75.8 ± 7.3 and 75.4 ± 8.0 (*p* = 0.872), respectively. The gender ratio (female/male) in the active control group was 8/8, and the treatment group was 12/4 (*p* = 0.144). Age and gender did not exhibit statistically significant differences between the active control and treatment groups.

### 3.2. BDNF Levels

**Figure 2:**
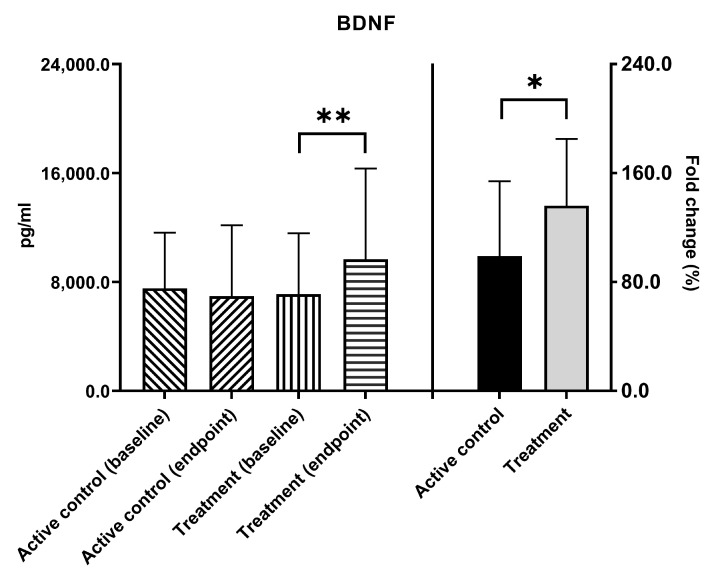
Serum BDNF levels were analyzed at baseline and after 12 weeks of probiotic intervention in active control and treatment groups of AD patients. BDNF = brain-derived neurotrophic factor; * *p* < 0.05, ** *p* < 0.01, indicating a significant difference either within or between groups.

AD is a multifaceted condition, with BDNF being just one of several factors whose levels are potentially related to the development of AD. The level of serum BDNF in AD patients was found to be lower than in healthy individuals [14,33]. After a 12-week intervention, the BDNF levels in the active control group did not show a significant change. However, it was observed that the BDNF levels in the treatment group increased significantly from a baseline value of 7115.1 ± 4461.9 pg/mL to an endpoint of 9678.5 ± 6652.9 pg/mL, with ** *p* = 0.005. Moreover, the fold change in BDNF levels in the treatment group exhibited a significant increase, compared to the active control group (136.3% vs. 98.5%, * *p* = 0.049, Figure 2).

### 3.3. Inflammatory Biomarkers, Cortisol Levels, and Antioxidant Capacity

**Figure 3:**
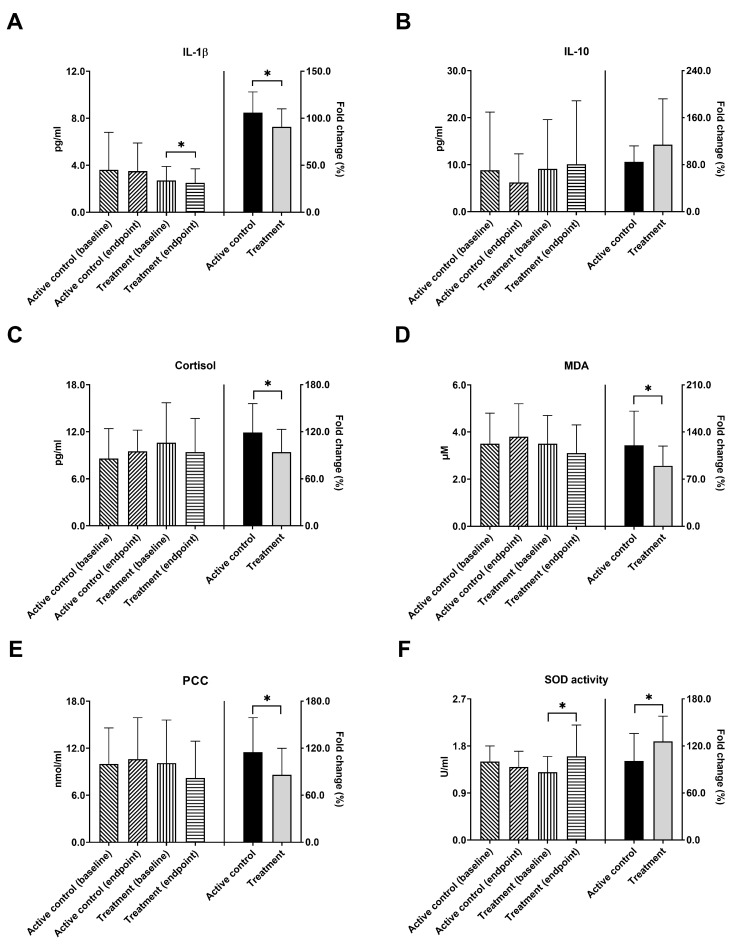
Probiotic supplements modulated inflammation, cortisol, and oxidative stress in AD patients. Serum levels of (**A**) IL-1β, (**B**) IL-10, (**C**) cortisol, (**D**) MDA, (**E**) PCC, and (**F**) SOD activity were analyzed at baseline and after 12 weeks of probiotic intervention in active control- and treatment-group AD patients. The values before and after the intervention were plotted on the left Y-axis, while the fold change was plotted on the right *Y*-axis. IL-1β = interleukin-1 beta; IL-10 = interleukin-10; SOD = superoxide dismutase; MDA = malondialdehyde; PCC = protein carbonyl content. * *p* < 0.05 indicates a significant difference either within or between groups.

In the pathological mechanisms of neurodegenerative diseases, the inflammatory process has been recognized as a significant factor in AD [34]. IL-1β functions as a pro-inflammatory cytokine, and IL-10 effectively suppresses the production of inflammatory cytokines. In Figure 3A, IL-1β levels in the treatment group are shown to have significantly decreased after the 12-week intervention (from 2.7 ± 1.2 to 2.5 ± 1.2 pg/mL, * *p* = 0.041). There was no significant change in the active control group. In terms of the fold change of IL-1β, a significant difference was found between these two groups. (106.1% vs. 90.7%, * *p* = 0.043). In Figure 3B, the levels of IL-10 showed no significant change upon comparing baseline and endpoint in both active control and treatment groups, and also no significant difference was seen between these two groups based on the fold change of levels. Furthermore, cortisol could enhance the toxic effects of pro-inflammatory cytokines on the hippocampus, thereby promoting the pathological development of AD [35]. No significant changes of cortisol levels were observed after intervention in both active control and treatment groups. The fold change of cortisol decrease was significantly larger in treatment group as compared with the active control group (119.4% vs. 94.3%, * *p* = 0.039, Figure 3C). Similar findings were found regarding the efficacy of the study probiotics on MDA and PCC activity. MDA, a byproduct of lipid peroxidation, and PCC, an indicator of protein oxidation, have both been established to be associated with aging and AD [36,37]. Although no significant decrease in these oxidative biomarkers was found in comparison between baseline and endpoint serum levels, fold changes of cortisol decrease were significantly larger in the treatment group than in the active control group (MDA,120.4% vs. 89.8%, * *p* = 0.046, Figure 3D, PCC, 115.0% vs. 85.9%, * *p* = 0.043, Figure 3E). Oxidative stress, caused by an imbalance between reactive oxygen species (ROS) and antioxidants, played a pivotal role in the progression of AD [36]. In Figure 3F, the antioxidant SOD activity in the treatment group significantly increased from the baseline of 1.3 ± 0.3 to 1.6 ± 0.6 U/mL (* *p* = 0.012) after intervention. Additionally, the fold change in SOD activity was significantly higher in the treatment group, compared to the active control group (100.6% vs. 126.1%, * *p* = 0.040).

### 3.4. Cognitive Function

**Figure 4:**
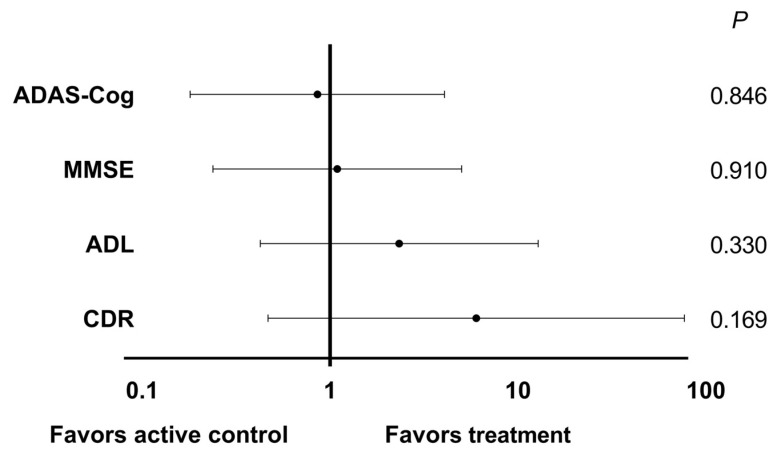
The ORs with 95% confidence intervals (95% CI) for the active control or treatment group in a 12-week intervention with probiotic supplements on AD assessment scales have been determined. These ORs with 95% CIs were used as dichotomous variables to assess whether there was a deterioration when comparing scores between before and after the intervention. ORs = odds ratios; ADAS-Cog = Alzheimer’s Disease Assessment Scale-Cognitive Subscale; MMSE = Mini-Mental State Examination; ADL = Activities of Daily Living; CDR = Clinical Dementia Rating.

The cognitive test scores (ADAS-Cog, MMSE, ADL, and CDR) of the active control and treatment groups during the intervention were presented in Table 1. There were no significant differences in these four assessment scales from baseline to the end of the 12-week intervention within the group. Additionally, the fold change of scores showed no significant difference in the active control group and probiotic group (data not shown): ADAS-Cog (105.4 ± 23.7 vs. 105.5 ± 27.5, *p* = 0.988); MMSE (101.8 ± 11.9 vs. 101.1 ± 12.9, *p* = 0.807); ADL (101.2 ± 11.6 vs. 95.8 ± 22.5, *p* = 0.400); CDR (112.5 ± 46.5 vs. 100.0 ± 31.6, *p* = 0.381). To assess the potential influence of age and gender on the effects of probiotic supplements on AD progression, a segmented analysis was conducted. Non-deterioration was defined as either an increase in or maintenance of scores in MMSE and ADL, or a decrease in or maintenance of scores in ADAS-Cog and CDR after the intervention. The age- and gender-adjusted ORs for ADAS-Cog (ORs = 0.9, 95% CI = 0.2 to 4.1, *p* = 0.846), MMSE (ORs = 1.1, 95% CI = 0.2 to 5.0, *p* = 0.910), ADL (ORs = 2.3, 95% CI = 0.4 to 12.8, *p* = 0.330), and CDR (ORs = 6.0, 95% CI = 0.5 to 76.9, *p* = 0.169) are presented in Figure 4. In each assessment scale, although there was no significant difference in the ORs, the treatment group exhibited a preference for non-deterioration in AD patients.

### 3.5. Changes in the Gut Microbiota Composition

**Figure 5:**
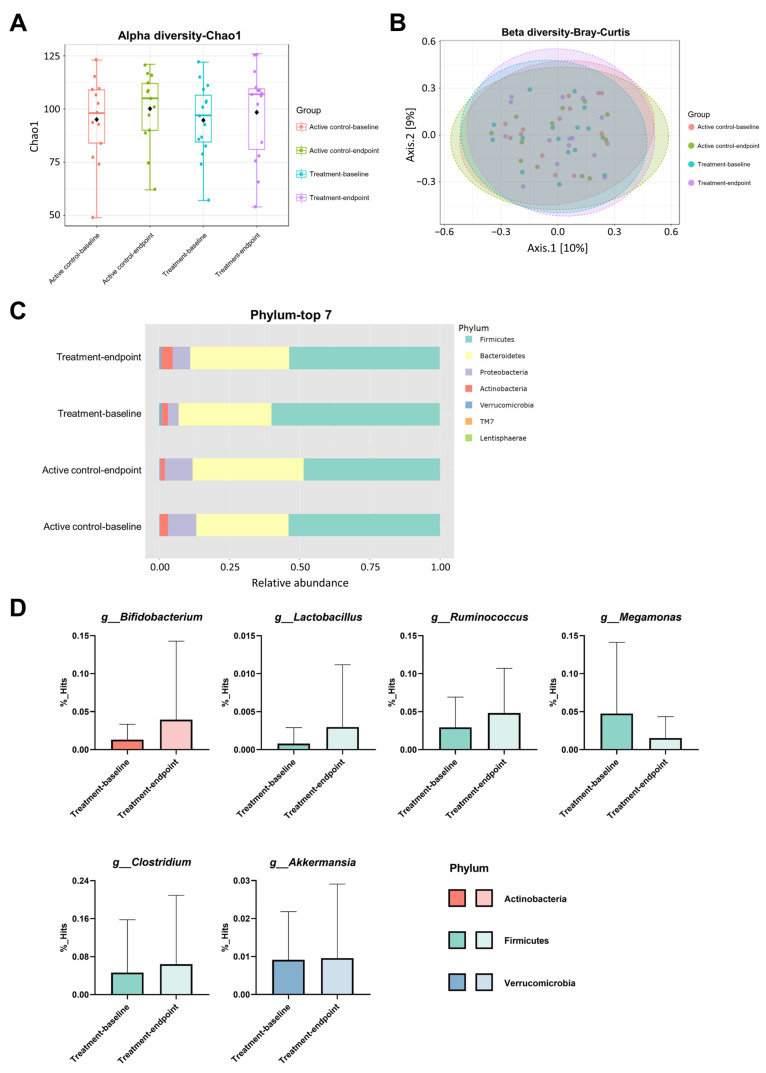
Gut microbiota diversity, the top 7 phyla, and altered genera after a 12-week probiotic intervention in AD patients were analyzed. (**A**) Alpha diversity, (**B**) beta diversity, (**C**) the composition of the top 7 most abundant phyla, and (**D**) the changed genera in the treatment group were examined.

To elucidate whether supplementing probiotics affects the gut microbiota composition in AD patients, the bacterial abundance indices Chao1 (α diversity index) and cluster similarity evaluated using Bray–Curtis (β diversity index) were analyzed at baseline and after a 12-week intervention. However, there were no significant differences observed in the Chao1 index (Figure 5A, *p* = 0.530) and Bray–Curtis (Figure 5B, *p* = 0.607) similarity within both the active control and treatment groups. The microbial compositions of the top seven most abundant phyla were compared in AD patients before and after the intervention (Figure 5C). After the 12-week intervention, the treatment group experienced an increase in the abundance of *Bifidobacterium* (1.3% to 3.9%, *p* = 0.317), *Lactobacillus* (0.1% to 0.3%, *p* = 0.354), *Ruminococcus* (2.9% to 4.8%, *p* = 0.286), *Clostridium* (4.6% to 6.4%, *p* = 0.321), and *Akkermansia* (0.9% to 1.0%, *p* = 0.934) at the genera level. Conversely, the presence of *Megamonas* (4.8% to 1.5%, *p* = 0.213) decreased in the treatment group.

## 4. Discussion

AD is a neurodegenerative disease that results in a decline in cognitive function, and as of now, there is no curative treatment [38]. In this study, we observed a significant increase in serum BDNF levels, a decreased level of inflammatory cytokine IL-1β, as well as an increased antioxidant SOD activity in the treatment group after a 12-week intervention in AD patients. In addition, greater benefit was found in the group that received a high dose of probiotic supplements than in the low-dose group. To the best of our knowledge, this is the first randomized, double-blinded, active-controlled trial on the efficacy of probiotic supplements specifically focusing on BDNF as well as various inflammatory and antioxidant biomarkers in the target population who have already been clinically diagnosed with Alzheimer’s dementia.

BDNF plays a pivotal role in regulating synaptic transmission, promoting neuronal plasticity, and preventing neuroinflammation and neuronal apoptosis. Serum BDNF is a biological indicator of cognitive function in the elderly [39]. The serum BDNF level in AD patients was found to be lower than that in healthy individuals [14]. Leyhe et al. reported that after 15 months of treatment with donepezil, serum BDNF levels in AD patients can be upregulated and show no significant difference compared to healthy individuals [40]. Kang et al. noted that consistent aquatic exercise in elderly women in the early stages of aging can elevate the expression of BDNF, contributing to the preservation and improvement of cognitive function. This elucidates the importance of early anti-aging measures and exercise in promoting the production of neurotrophic factors [41]. Probiotics, whether utilized as food additives or fermentation microorganisms, have gradually become a common supplement for people’s life and healthcare. It has been reported that the oral consumption of *Lactobacillus plantarum* DW2009 can elevate BDNF levels in the serum, contributing to improved cognitive function [42]. Coincidentally, a clinical study focusing on elderly residents in the community has also confirmed that the significant increase in serum BDNF is evident after 12 weeks of probiotic consumption [43]. In this study, we observed a significant increase in serum BDNF levels in the treatment group after the 12-week intervention, and the fold change in BDNF is higher than the active control group. This suggests that probiotic supplements can improve BDNF expression and may have the potential to enhance cognitive function in AD patients.

Pro-inflammatory cytokines play a significant role in central nervous system injuries and neurodegenerative diseases, including AD [44,45,46]. The immune system’s inflammatory process may lead to the development of neurodegenerative diseases through the excessive phosphorylation of TAU protein [47]. Research indicates a bidirectional relationship between the expression of BDNF and the regulation of neuroinflammation, and that IL-1β can reduce the activity of BDNF through the PI3-K pathway, thereby decreasing BDNF’s neuroprotective abilities [48]. Elevated levels of the pro-inflammatory cytokine IL-1β have been observed in the brains of AD patients [49]. Our data indicate that probiotic supplements were shown to downregulate IL-1β compared to the active control group. It may be suggested that probiotics may regulate the inflammatory factor IL-1β, leading to the upregulation of serum BDNF.

Cortisol is a hormone believed to regulate chronic stress, and it is known that cortisol levels are higher in AD patients, which may be a risk factor for cognitive degeneration [50,51,52]. A probiotic mixture of *Lactobacillus* and *Bifidobacterium* can effectively reduce cortisol levels and modify brain metabolites in aging rats [53]. In this study, although the cortisol levels did not exhibit significant changes during the intervention, the fold change of cortisol decrease in the treatment group was greater than in the active control group.

Oxidative stress is closely related to AD, as it fosters the accumulation of β-amyloid (Aβ) protein, a hallmark of AD pathogenesis [45,54,55,56,57]. Reactive oxygen species also alter neuronal lipid molecules, leading to changes in membrane properties and the formation of lipid peroxidation byproducts, such as 4-hydroxynonenal or MDA. Oxidation processes also affect proteins by adding carbonyl groups [55]. AD patients often exhibit elevated levels of lipid peroxidation and protein carbonyls, indicating increased oxidative stress [58]. Probiotic supplements have been shown to reduce inflammation, increase SOD, and lower oxidative stress [59]. Kaushal and Kansal noted a significant reduction in plasma PCC in mice aged 12 months upon the administration of probiotic Dahi containing *Lactobacillus acidophilus* and *Bifidobacterium bifidum* for a duration of 4 months [60]. Similarly, Our previous study has shown that probiotic supplements reduced the MDA levels in the brain of aging mouse models [17]. Considering these findings, specific probiotics demonstrate robust and effective antioxidant capabilities. A multi-strain probiotic containing BLI-02, Bv-889, CP-9, VDD088, and PL-02 was identified as a comprehensive antioxidant probiotic combination. The results of the present study support the evidence of probiotics in enhancing SOD activity and reducing peroxidation markers, such as MDA and PCC, after a 12-week intervention in AD patients.

Research has indicated that supplementation with *Bifidobacterium breve* MCC1274 for 24 weeks can enhance cognitive function and inhibit the progression of brain atrophy in elderly patients with mild cognitive impairment (MCI) [61]. In this study, although there were no significant improvements in cognitive function assessed via ADAS-Cog, MMSE, ADL, and CDR after the 12-week intervention, the scores of the treatment group showed a trend of lowering the rate at which AD symptoms worsen. A short period of intervention and a small sample size are probably some of the reasons for the clinically non-significant results. Further randomized controlled trials with longer administration time are needed to confirm the effect of probiotic supplements on AD patients.

The relationship between gut microbiota and Alzheimer’s disease is not fully understood. An imbalance in gut microbiota can lead to reduced levels of BDNF in the hippocampus and cortical midbrain, potentially inducing cognitive impairment through the gut–brain axis [62]. Multiple reports have shown that bacterial diversity is decreased in AD patients, with reductions in Firmicutes and *Bifidobacterium*, and an increase in Bacteroidetes [63,64]. Additionally, there is an observed increase in Actinobacteria [65]. Research suggests that higher levels of *Clostridium* are associated with better cognitive performance [66]. Butyrate-producing bacteria, such as *Ruminococcus* and *Clostridium*, have been verified to metabolize unprocessed carbohydrates, resulting in the generation of short-chain fatty acids (SCFAs) and lactic acid within the intestine [67]. SCFAs are also thought to modulate the effects of BDNF in the central nervous system [68]. This metabolic process supports the proliferation of *Lactobacillus* and *Bifidobacterium* [67]. Another study indicated that *Akkermansia* slowed down the pathological changes in the brains of AD model mice and alleviated spatial learning and memory impairment [69]. In our previous study, we have observed that the probiotic supplements increased the *Bifidobacterium*, *Lactobacillus*, and *Akkermansia* of gut microbiota in aging mice [17]. The present study revealed that the gut microbiota of the treatment group exhibited slight changes with *Bifidobacterium*, *Lactobacillus*, *Ruminococcus*, *Clostridium*, and *Akkermansia* at the genera level, all of which increased while *Megamonas* decreased. However, these changes at the phyla and genera levels were not significantly different after the 12-week intervention, and the diversity in alphas and betas between the active control and treatment groups after the intervention showed no significant differences. Several factors can affect human microbiota, such as diet, environment, medication, and mood. The small number of participants may also lead to an insignificant result of this short-term intervention.

The present study is not without limitations. First, the diagnosis of AD in our participants was based on DSM-V, or NINCDS-ADRDA Alzheimer’s Criteria, or the 2011 NI-AAA guidelines, without AD biomarker confirmation. According to the updated NI-AAA guideline in 2018 [70], the diagnosis of AD has been toward biological definition in recent years. Individuals with evidence of brain deposits with abnormal protein amyloid plaque or TAU, found via positron emission tomography imaging or through examining the cerebrospinal fluid or blood, can be diagnosed with AD. Whether they are preclinical or symptomatic, AD is based on the presence of cognitive impairment. When this study started to enroll patients in 2020, we did not use these AD biomarkers to make the diagnosis, despite the fact that all the participants in this study previously underwent comprehensive medical evaluation, including typical clinical presentation, mental test, blood test, and neuroimaging to exclude other dementia diseases. Without the confirmation of an AD biomarker (image or fluid), the diagnosis of AD was performed by investigating neurologists according to the aforementioned criteria but which did not fully meet the updated 2018 NI-AAA guidelines. Second, our last assessment was performed at week 12 without a follow-up of the subsequent changes of microbes after the participants stopped taking the study probiotics. For a better exploration of the efficacy of these probiotic supplements, studies with a longer duration of follow-up and a repeated assessment of the fecal microbes after patients have completed their supplement intake are needed in the future.

## 5. Conclusions

The findings of this clinical trial, even with a short period of intervention in a small target population sample size, provide evidence of the positive impacts of probiotics in AD patients. We conclude that, with sufficient doses and a proper formulation of probiotic supplementation, serum BDNF can be enhanced along with a reduction in oxidative stress and an increase in antioxidants among dementia patients who have already been clinically diagnosed with AD. Probiotics might have potential benefits to slow down the decline in cognitive function among symptomatic AD patients. Further studies with a larger sample size and longer intervention durations are needed to confirm the clinical benefit of probiotics.

## Figures and Tables

**Table 1:** The examined age, gender, and baseline and endpoint cognitive functions of AD patients.

	Active Control Group			Treatment Group		^a^ *p*-Value
Age (years)	75.8 ± 7.3			75.4 ± 8.0		0.872
Gender (female/male)	8/8			12/4		0.144
AD assessment scales	Baseline	Endpoint	^b^ *p*-value	Baseline	Endpoint	^b^ *p*-value
ADAS-Cog	21.3 ± 8.3	21.6 ± 7.7	0.711	25.9 ± 12.8	26.2 ± 10.8	0.768
MMSE	21.1 ± 3.8	21.4 ± 4.4	0.596	18.8 ± 4.5	18.9 ± 4.8	0.833
ADL	62.4 ± 12.3	62.7 ± 11.8	0.871	53.9 ± 12.7	51.0 ± 16.3	0.363
CDR	0.7 ± 0.3	0.8 ± 0.4	0.497	1.0 ± 0.5	1.0 ± 0.5	0.751

Data are expressed as mean ± standard deviation. ^a^: The difference between the active control group and treatment group. ^b^: The difference between the baseline and endpoint. Abbreviations: ADAS-Cog = Alzheimer’s Disease Assessment Scale-Cognitive Subscale; MMSE = Mini-Mental State Examination; ADL = Activities of Daily Living; CDR = Clinical Dementia Rating.

## Data Availability

The datasets used and/or analyzed during the current study are available from the corresponding author on reasonable request.
